# M-Track: A New Software for Automated Detection of Grooming Trajectories in Mice

**DOI:** 10.1371/journal.pcbi.1005115

**Published:** 2016-09-16

**Authors:** Sheldon L. Reeves, Kelsey E. Fleming, Lin Zhang, Annalisa Scimemi

**Affiliations:** 1 Department of Computer Science, State University of New York Polytechnic Institute, Utica, New York, United States of America; 2 Department of Biology, State University of New York Albany, Albany, New York, United States of America; 3 Department of Computer Science, State University of New York Albany, Albany, New York, United States of America; Universite de Montreal, CANADA

## Abstract

Grooming is a complex and robust innate behavior, commonly performed by most vertebrate species. In mice, grooming consists of a series of stereotyped patterned strokes, performed along the rostro-caudal axis of the body. The frequency and duration of each grooming episode is sensitive to changes in stress levels, social interactions and pharmacological manipulations, and is therefore used in behavioral studies to gain insights into the function of brain regions that control movement execution and anxiety. Traditional approaches to analyze grooming rely on manually scoring the time of onset and duration of each grooming episode, and are often performed on grooming episodes triggered by stress exposure, which may not be entirely representative of spontaneous grooming in freely-behaving mice. This type of analysis is time-consuming and provides limited information about finer aspects of grooming behaviors, which are important to understand movement stereotypy and bilateral coordination in mice. Currently available commercial and freeware video-tracking software allow automated tracking of the whole body of a mouse or of its head and tail, not of individual forepaws. Here we describe a simple experimental set-up and a novel open-source code, named M-Track, for simultaneously tracking the movement of individual forepaws during spontaneous grooming in multiple freely-behaving mice. This toolbox provides a simple platform to perform trajectory analysis of forepaw movement during distinct grooming episodes. By using M-track we show that, in C57BL/6 wild type mice, the speed and bilateral coordination of the left and right forepaws remain unaltered during the execution of distinct grooming episodes. Stress exposure induces a profound increase in the length of the forepaw grooming trajectories. M-Track provides a valuable and user-friendly interface to streamline the analysis of spontaneous grooming in biomedical research studies.

This is a *PLOS Computational Biology* software paper.

## Introduction

Grooming is an evolutionary ancient innate behavior performed by most vertebrate species, including humans [1, 2]. In mice, self-grooming is one of the most frequently observed activities in the motor repertoire, representing >15% of the mice waking time [3]. Each grooming episode consists of predictable and stereotyped movements that mice execute as they groom along the rostro-caudal axis of their body [4, 5]. The micro-structure of complete grooming episodes consists of four major identifiable phases, during which mice lick their forepaws (Phase I), rub their face (Phase II), their body (Phase III), hind legs (Phase IV) and tail and genitals (Phase V) [6, 7]. Each phase can be scored to determine the consistency with which different types of strokes are performed [5, 6, 8–10]. The neuronal circuits that control grooming are mainly located in the basal ganglia nuclei. Therefore, changes in grooming features like the speed of the forepaw movement and the order with which mice transition between distinct grooming phases can provide information on alterations in the functional properties of basal ganglia circuits [11–15]. Accordingly, dysregulated activity of basal ganglia circuits in animal models of neuropsychiatric disorders (e.g. obsessive compulsive disorder, Tourette’s syndrome) show a disrupted micro-structure of grooming episodes [5, 16, 17]. These findings indicate that developing accurate tools to detect grooming could significantly improve our ability to monitor functional changes in specific neuronal circuits that control grooming, thereby providing new important information for behavioral studies in animal models of neurological and neuropsychiatric disorders.

Traditional grooming analysis is performed on episodes evoked by exposing mice to novelty cues, mild and strong stressors (e.g. light exposure, water sprays, forced swim tests, restraint stress [18, 19]). The analysis of these evoked grooming episodes is still performed manually and relies on visual monitoring and manual scoring of each episode [5, 8, 20]). Admittedly, these approaches are prone to caveats. *First*, the structure of stress-induced grooming episodes may not be entirely representative of that of spontaneous grooming episodes. Therefore their predictive value for actual changes in the activity of basal ganglia circuits and their involvement in the onset of brain disorders may be limited [8]. *Second*, manual scoring of patterned strokes is subjective and may be inaccurate particularly when mice transition between distinct grooming phases rapidly, introducing artifacts for the analysis, data reproducibility and interpretation.

A number of video-tracking software has become available through commercial and public sources, to automate motion detection and analysis in different behavioral tests, in rodents, zebrafish, honey bees and flies (e.g. Noldus EthoVision, Stoelting AnyMaze, JAABA [21], ZebraZoom [22], CTRAX [23], K-Track [24], etc.). Video-tracking analysis of kinematically normal prehension in head-restrained mice has also been recently developed [25]. None of the currently available software, however, is specifically designed or can be easily implemented for automated tracking both forepaws during spontaneous grooming in multiple freely-behaving mice. The operating algorithms of many of these software are based on color-detection or shape-recognition criteria. These criteria cannot be used, on their own, to unambiguously detect the movement of the forepaws. In mice, the left and right forepaws have the same color (creating ambiguity for color detection) and their shape changes continuously over time as mice extend or flex their digits (creating ambiguity for shape detection). The two forepaws often overlap during grooming and since they may have the same natural color and shape, it is difficult for an algorithm to distinguish between each one of them. Machine-learning and robust cascade pose regression methods can potentially overcome these limitations and have been used to track and classify different types of movement in multiple flies in flat behavioral arenas [21] and to track the movement of one single forepaw in head-restrained mice [25, 26]. A potential weakness of these approaches is that they require the tracked item (i.e. the forepaw) to be always present in the field of view and never overlap with other structures with similar features. This can pose problems in the analysis of spontaneous grooming, because the forepaws can temporarily disappear from the field of view when mice rub behind their ears and head, for example. To overcome these limitations, we assembled a simple experimental set-up and developed novel open-source software based on the use of a probabilistic-programming algorithm, which allows us to perform kinematic tracking of forepaw movements during spontaneous grooming episodes in multiple freely-moving mice. This software can be used to unambiguously identify and track forepaw trajectories and measure movement stereotypy, bilateral coordination and the length of grooming trajectories. It can be used to track forepaw movement during specific grooming phases and also during the execution of other types of movements in behavioral studies (e.g. walking), providing a valuable tool for the behavioral neuroscience research community.

## Design, Implementation and Results

### Experimental apparatus and acquisition overview

**Fig 1:**
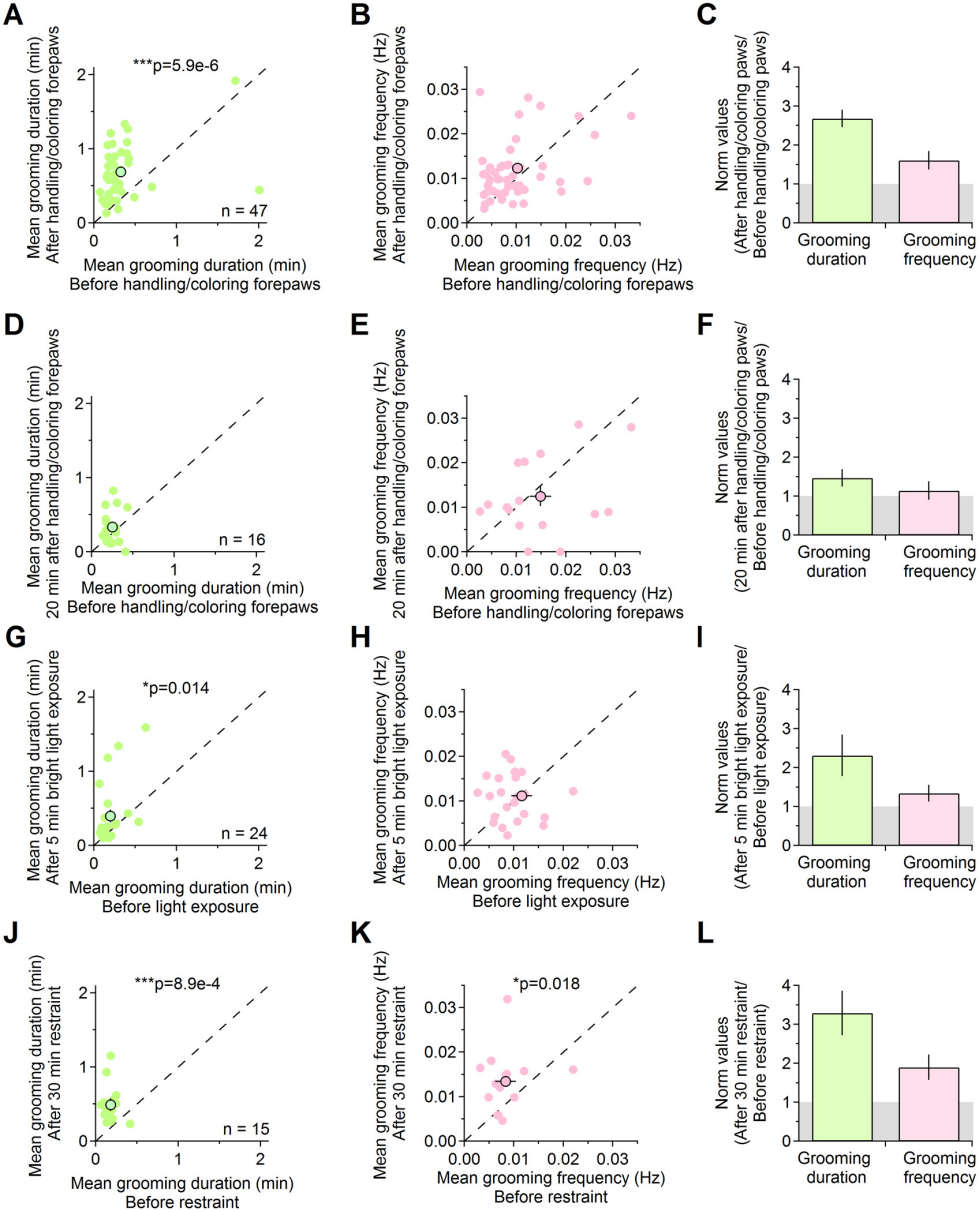
Sensitivity of grooming to mild and strong stressor exposure. **(A)** Relationship between the mean duration of grooming episodes in mice tested before and immediately after being handled to color their forepaws (before: 0.32±0.05 min, after: 0.69±0.07 min (n = 47), ***p = 5.9e-6). **(B)** Relationship between the mean frequency of grooming episodes in mice tested before and immediately after handling/coloring the forepaws (before: 0.010±0.001 Hz, after: 0.012±0.001 Hz (n = 47), p = 0.051). **(C)** Relative change in the duration and frequency of grooming episodes in mice tested immediately after being handled to color their forepaws (duration: 2.68±0.22, frequency: 1.61±0.23 (n = 47)). **(D)** Relationship between the mean duration of grooming episodes in mice tested before and 20 min after being handled to color their forepaws (before: 0.25±0.02 min, after 0.33±0.06 min (n = 16), p = 0.098). **(E)** Relationship between the mean frequency of grooming episodes in mice tested before and 20 min after handling/coloring the forepaws (before: 0.015±0.002 Hz, after: 0.012±0.002 Hz (n = 16), p = 0.582). **(F)** Relative change in the duration and frequency of grooming episodes in mice monitored 20 min after handling the mice to color their forepaws (duration: 1.47±0.26, frequency: 1.14±0.23 (n = 16)). **(G)** Relationship between the mean duration of grooming episodes in mice tested before and after 5 min exposure to bright light (before: 0.20±0.03 min, after: 0.40±0.08 min (n = 24), *p = 0.014). **(H)** Relationship between the mean frequency of grooming episodes in mice tested before and after 5 min bright light exposure: 0.012±0.002 Hz, 0.011±.001 Hz (n = 24), p = 0.636). **(I)** Relative change in the duration and frequency of grooming episodes in mice exposed to bright light (duration: 2.31±0.54, frequency: 1.34±0.21 (n = 24)). **(J)** Relationship between the mean duration of grooming episodes in mice tested before and after restraint stress (before: 0.18±0.02 min, after: 0.49±0.07 min (n = 15), ***p = 8.9e-4). **(K)** Relationship between the mean frequency of grooming episodes in mice tested before and after restraint stress: 0.008±0.001 Hz, 0.013±0.002 Hz (n = 15), *p = 0.018). **(L)** Relative change in the duration and frequency of grooming episodes induced by 30 min restraint stress (duration: 3.29±0.57, frequency: 1.90±0.33 (n = 15)).

In order to unambiguously track individual forepaws, we handled mice to label the back and front sides of their forepaws with 2–4 layers of fluorescent permanent markers (Sharpie Neon green and Neon magenta). Handling mice is a non-invasive routine manipulation for husbandry, but it might trigger a mild stress response that disrupts the spontaneous grooming behavior of mice [27]. To determine whether the procedure of handling mice and coloring their forepaws induces a stress response that affects grooming, we measured the duration and frequency of grooming episodes before and after handling the mice and coloring their forepaws (Fig 1). The duration, not the frequency, of grooming episodes in mice tested immediately after coloring the forepaws was increased with respect to the one measured before handling the mice (Fig 1A–1C; grooming episode duration before and immediately after handling/coloring the forepaws: 0.32±0.05 min, 0.69±0.07 min (n = 48), ***p = 5.9e-6; grooming frequency before and immediately after handling/coloring the paws: 0.010±0.001 Hz, 0.012±0.001 Hz (n = 48), p = 0.051). This effect was no longer detected in mice tested 20 min after being handled (Fig 1D–1F; grooming episode duration before and 20 min after handling/coloring the forepaws: 0.25±0.02 min and 0.33±0.06 min (n = 16), p = 0.098; grooming frequency before and 20 min after handling/coloring the forepaws: 0.015±0.002 Hz and 0.012±0.002 Hz (n = 16), p = 0.582). These results suggest that handling mice to color their forepaws induces a mild stress response and a transient increase in the duration of grooming episodes, an effect that disappears in 20 min. We performed additional experiments to compare the transient effect on grooming induced by handling mice to color their forepaws with the effect of exposing mice to known mild and strong stressors. We used a brief (5 min) exposure to direct bright light (14,000 lux) as a mild stressor (Fig 1G–1I). We confirmed that this experimental protocol did not induce any heat stress in mice, because the temperature of the behavioral arena remained constant (20°C) over the course of the 5 min bright light exposure. Similarly to handling/coloring the forepaws, brief exposure to bright light induced a significant increase in the duration, not the frequency, of grooming episodes (grooming episode duration before and after exposure to bright light: 0.20±0.03 min, 0.40±0.08 min (n = 24), *p = 0.014; grooming frequency before and after exposure to bright light: 0.011±0.002 Hz, 0.011±.001 Hz (n = 24), p = 0.636). Subjecting mice to a 30 min restrain protocol, a known strong stressor, induced a pronounced increase in both the grooming episode duration and frequency (Fig 1J–1L; grooming episode duration before and after restraint stress: 0.18±0.02 min, 0.49±0.07 min (n = 15), ***p = 8.9e-4; grooming frequency before and after restraint stress: 0.008±0.001 Hz, 0.013±0.002 e-3 Hz (n = 15), *p = 0.018). Taken together, these results indicate that the effect on grooming induced by coloring the mice forepaws is similar to that induced by exposing mice to mild stressors (e.g. brief bright light exposure), not strong stressors (e.g. restraint). The effect is transient and disappears in 20 min. Based on these findings, we performed our analysis on videos acquired 20 min after coloring the mice forepaws and we suggest users to use a similar experimental design in studies that aim to analyze the spontaneous, as opposed to stress-induced grooming in mice.

**Fig 2:**
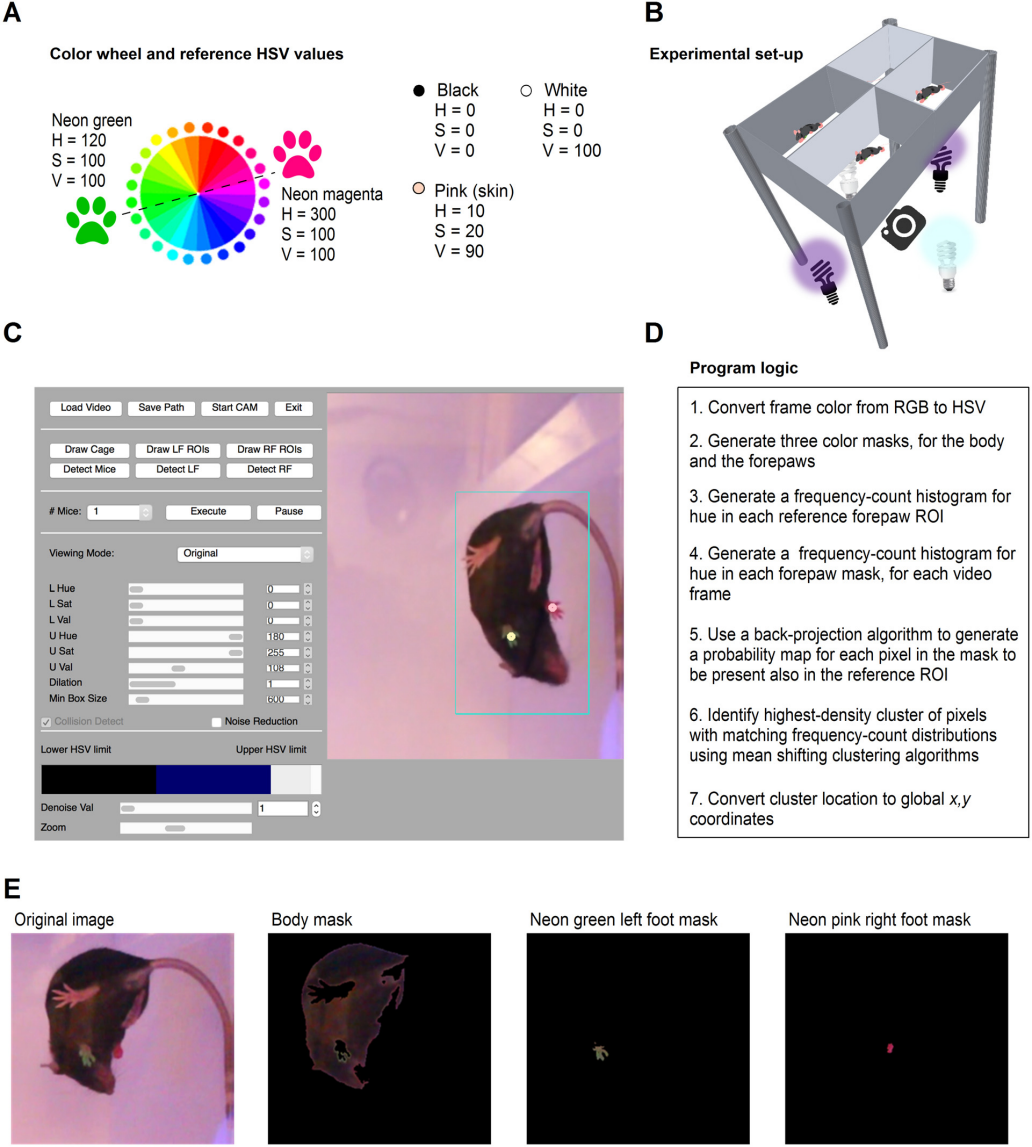
Experimental set-up and algorithm description. **(A)** Hue, saturation and value (HSV) color wheel used to select complementary colors for labeling the mouse forepaws. Complementary colors, like the ones we chose to mark the forepaws of mice in our own experiments (Neon green and Neon magenta) are located on opposite sides of the HSV color wheel. Neon green and Neon magenta are well suited to perform trajectory analysis with M-Track because their hue is different from each other and from the hue of the skin and of the fur of black or white mice. **(B)** Schematic representation of the behavioral apparatus used to acquire videos of spontaneous grooming in multiple freely-behaving mice. The grooming chamber has thin, clear floor and white walls and consists of four adjacent enclosures. All grooming episodes are recorded using a video camera positioned 12” below the behavioral arena. **(C)** Outline of M-Track GUI. The GUI allows the user to load a video file and draw the perimeter of the behavioral arena and the ROIs for the left and right forepaws, completely labelled with Neon green and Neon magenta permanent markers. The detected forepaws are labelled in the GUI with colored dots. The ROI for the mouse body (turquoise box) is used by M-Track to identify the location of the two forepaws. **(D)** Sequence of analytical operations performed by the M-Track algorithm during video-tracking analysis. **(E)** Example representations of body and foot masks generated by varying the HSV settings for the mouse body and left/right foot in the M-Track GUI. The first image on the left represents a non-processed snapshot of a mouse with labelled forepaws.

The selection of colors used to label the mice forepaws and monitor their trajectories during grooming is at complete discretion of the user. We recommend to choose complementary colors, because they create a strong contrast when placed close to each other (Fig 2A). In addition, it is important that the hue of the selected colors is different from the hue of other colors present in the behavioral arena. This is due to the fact that the algorithm that M-Track uses to locate the forepaws is largely based on the detection of pixels with unique hue. After extensive tests on different colors (e.g. orange, blue, etc.), we selected Neon green and Neon magenta as the labeling colors that worked best in our hands. Accordingly, the hue of Neon green (H = 120) and Neon magenta (H = 300) is very different from that of the skin (H = 10) and fur of black and white mice (H = 0) like C57BL/6 and Swiss Webster, which we used to test out software and are commonly used in neuroscience research. To perform the experiments, we positioned four C57BL/6 mice in four adjacent enclosures (6”H x6”W x10”L), with white walls and lid, and a transparent floor (Fig 2B). We used black and white lights to illuminate the entire behavioral arena (enclosure illuminance = 20–50 lux). Enclosures with similar dimensions but with black walls and lid were used when testing Swiss Webster mice, which have white fur (enclosure illuminance = 80–220 lux). Neon colors glow under black light illumination, and this significantly improves the accuracy of forepaw detection during video-tracking analysis, at times when both forepaws are visible and when any of them is hidden by the mouse body. A color-sensitive camera was positioned 12” below the surface of the behavioral arena and was used to acquire 10 min long video files (.MOV,.MP4, ~4 GB) using slow-motion camera settings (60 fps), to accurately track the movement of the forepaws in consecutive frames. In this experimental layout, the white light sources used to illuminate the behavioral arena ensured high levels of background illumination during slow-motion video acquisition, which is necessary due to the limited amount of light collected by the camera at high acquisition frame rates. Finer adjustments in the intensity of the background illumination can be performed, but they are not crucial as they lead to changes the image saturation, not hue (the parameter that M-Track uses to calculate the frequency-count histogram for the back projection algorithm, to detect the position of the forepaws in each video frame).

### Overview of the graphical user interface

The M-Track graphical user interface (GUI) allows the user to load a video file and define HSV color detection parameters for each forepaw (Fig 2C). Once loaded, the M-Track GUI displays the first frame in the acquired video file, which is the one used to optimize the HSV color detection parameters before starting the analysis. In its current implementation, M-Track can track the trajectory of the forepaws in behavioral arenas composed of one, two or four adjacent enclosures organized as 1x1 (i.e. one mouse), 1x2 (i.e. two mice) or 2x2 arrays (i.e. four mice). The code can be easily implemented to increase the number of tracked enclosures and perform higher throughput analysis. After loading the video and selecting the number of mice to be analyzed, with three consecutive click-and-drag steps, the user sets the *x*,*y* position for three of the four corners of the enclosure in which the trajectory analysis needs to be performed. M-Track identifies this as the outer margins of the region in which the mouse can move. M-Track rotates the displayed image to correct for potential inaccuracies in the relative position of the video camera and the behavioral arena during video acquisition and the user can zoom in and out the selected window with a slider, to visualize finer details of the cropped image. When analyzing multiple mice, the user is required to draw lines along the walls of the behavioral arena that separate adjacent enclosures (i.e. one line when analyzing two mice in a 1x2 array; two lines crossing each other when analyzing four mice in a 2x2 array). Through the M-Track GUI, the user sets the lower and upper limits for HSV values, to define the color masks for the body (an important step when analyzing multiple mice simultaneously). As a next step, the user clicks and drags the left button of the computer mouse to draw the region of interest (ROI) for the left and right forepaws. This is done by clicking the left mouse button on the top left corner of the ROI and by releasing it on the opposite, bottom right, corner of the ROI. When this is done, M-Track calculates and displays the HSV parameters for the ROIs on the top left corner of the GUI, to provide users with guideline values for setting the upper and lower HSV limits for the forepaw detection (see below). It is important to correctly position these two ROIs within—not around—the colored portion of the left and right forepaws. This is because the HSV profile of the pixels contained within these two ROIs are the ones that the M-track detection algorithm uses to identify the location of the forepaws in each video frame. Once the ROIs have been drawn, through the M-Track GUI, the user sets the lower and upper limits for the HSV values, to define the color masks for the forepaws. This operation allows the user to filter out all other colors except the ones of the mouse fur (for the mouse body mask) and the colored forepaws (for the two forepaws masks). M-Track displays in real time the effect of changing the upper and lower HSV limits for the mouse body and forepaws color masks, allowing the user to quickly optimize the detection settings. The colors corresponding to the selected lower and upper HSV limits are also shown in real time, in the bottom section of the GUI. M-Track uses the body color mask to generate a mouse body ROI (displayed as a turquoise mouse bounding box in the M-Track GUI), which is used to determine which mouse the tracked forepaws belong to. The forepaws are tracked only if they fall within an area that is 10% larger than the mouse body ROI. The same HSV limits are used to detect the body and forepaws when analyzing multiple mice in the same video. In our experimental conditions, all enclosures had similar levels of illumination. Changes in the brightness of different enclosures do not compromise the analysis because they largely affect saturation, whereas the M-Track analysis is largely based on the analysis of hue. The user can also select to enlarge each mask using the “Dilation” command. The “Min Box Size” sets a lower threshold for the size of the pixel cluster that identifies the position of each forepaw, preventing M-Track from erroneously attributing the forepaw location to individual pixels with the same HSV profile of the one measured in the ROIs. Once the ROIs are drawn and the HSV settings are selected, the user selects the directory where the output file is saved and specifies the name and extension of the output file (.TXT,.XML,.CSV or.HTML). M-Track uses two colored dots to display the position of the forepaws and stores information on the *x*,*y* coordinates of the left and right forepaws, the position of the center of mass of the mouse body and its orientation in each video frame in the output file. The tracking can be paused at any point during the analysis and the user can change the number of mice to track without having to reset all the HSV limits.

### Software description

M-Track is an open-source video-tracking algorithm that uses the Python programming language, the OpenCV library and the Qt4 framework. The motion trajectory analysis of the forepaws is performed using an object detection method based on color filtering and probabilistic models. The tracking method relies on color filtering, back projection algorithms and clustering analysis within the forepaws ROIs. The logics of the M-Track algorithm is summarized in Fig 2D. *First*, M-Track converts every frame in the video from the BGR (“Blue, Green, Red”; i.e. the convention byte order for OpenCV) to the HSV (“Hue, Saturation, Value”) color space. *Second*, it generates a background-subtracted color mask of the entire image according to the HSV settings of the mouse body and forepaws selected by the user in the GUI, to filter out the color of the fur and of the colored forepaws from that of the background of the video (Fig 2E). This approach for background subtraction is particularly useful: it simplifies the experimental design, because it does not require the experimenter to acquire, register and subtract from the video any arbitrary number of frames of the empty behavioral arena. The HSV color mask defined by the user is converted into a binary image, which is used to generate a contour of the mouse body. Analogous color masks based on the selected HSV limits are used to isolate the color of the forepaws from all other colors in the video and generate binary images. In each video frame, the size of the mouse body ROI (displayed as a turquoise box) is scaled based on the width and height of the body color mask in the binary image. The *x*,*y* coordinates of the center point of the mouse body color mask and ROI are stored in the output file generated by the software. M-Track detects the position of the forepaws within a region that is 10% larger than the mouse body ROI, to ensure that the forepaws are always tracked even if the mouse stretches one of the forelimbs away from the body. This features makes M-Track a versatile tool, which can accurately track the position of the forepaws in mice regardless of their size and age. It also allows M-Track to identify which mouse the forepaws belong to regardless of how close the mice are to each other. M-Track uses collision detection, which allows accurate detection of the mouse body even when its masked image is fragmented. The color mask for the forepaws is used to determine the position of the forepaws ROIs in each frame with a mean shift detection algorithm (see below). The current release of M-Track is exclusively based on color detection, not on pattern recognition, because the shape of the forepaws can change significantly as mice extend their digits and close their forepaws repeatedly during grooming. *Third*, M-Track computes a frequency-count histogram for the hue distribution, not saturation or value, in the first video frame, in each of the two forepaw ROIs. This approach improves the processing time of the algorithm and the detection accuracy, because the hue values do not change under different lighting conditions that may occur when the mouse moves in different regions of the enclosure or as mice lift their forepaws from the transparent floor of the behavioral arena. *Fourth*, M-Track generates a histogram of hue distribution for the new forepaws masks in consecutive frames. The back projection algorithm compares the original ROI histogram with that in the masks of the current frame and M-Track generates a probability map distribution representing the probability that each pixel in the mask of the new frame is also present in the original reference ROI. *Fifth*, by using a mean shifting clustering algorithm, M-Track identifies the highest-density cluster of pixels with the highest probability values. The position of this cluster is used to re-set the position of the ROI. OpenCV's Continuously Adaptive Mean (CAM) shift implementation uses the mean shift algorithm with track window scaling to resize the ROI according to the size of the pixel cluster: the bigger the pixel cluster, the bigger the ROI. When either of the two forepaws is lost from the camera field of view, M-Track increases progressively the size of the forepaw ROI until it matches the size of the body ROI and therefore easily tracks back the position of the forepaw as it reappears in the field of view. *Lastly*, M-Track converts the location of the pixel cluster with the highest similarity to that in the mouse body reference window into global *x*,*y* coordinates and stores them in the output file. It is the list of these global coordinates that is ultimately used to plot the *x*,*y* position and define the trajectory of the mouse forepaws for the duration of the video (in pixels). The user determines the pixel size using his/her own recording apparatus and converts pixel measurements of distance into measurements of distance in other units.

### Software detection accuracy

The ability of M-Track to accurately report the position of the mouse forepaws relies on the fact that: *(1)* the forepaws are labelled with colors with a unique hue, that is not found in the background or in the body of the animal in any video frame; *(2)* the user accurately constrains the HSV upper and lower limits to generate the body and forepaws color masks; *(3)* the users accurately positions the forepaws ROIs within the colored forepaws. M-Track can be used to analyze videos with non-uniform background, as long as the colors used to label the forepaws are different from each other and from any other color present in the background of each video frame. In our hands, the colors that work best to label the forepaws are Neon green and Neon magenta, because their hue is different from one another and from that of any other color present in our videos. Accordingly, the hue of Neon green (H = 120) and Neon magenta (H = 300) is very different from the hue of the mouse skin (H = 10) and fur (H = 0). M-Track continues to track the forepaws even when the mice lift them from the floor of the behavioral enclosure, as long as they remain visible within the field of view and the depth of field of the camera (8.5–13.4 cm in our experiments) and are not hidden by other parts of the mouse body. Based on our experience, mice can raise their forepaws <5 cm from the floor of the behavioral arena, so we recommend the use of cameras with a depth of field >5 cm, to ensure the forepaws are always tracked by the software. In some experimental conditions, the color of the forepaws may appear less bright as the forepaws are lifted from the floor of the behavioral arena. In the HSV color space, this corresponds to a change in saturation for the color of the forepaws, not in their hue. The M-Track algorithm is not affected by changes in saturation, because the back-projection algorithm compares hue distributions.

**Fig 3:**
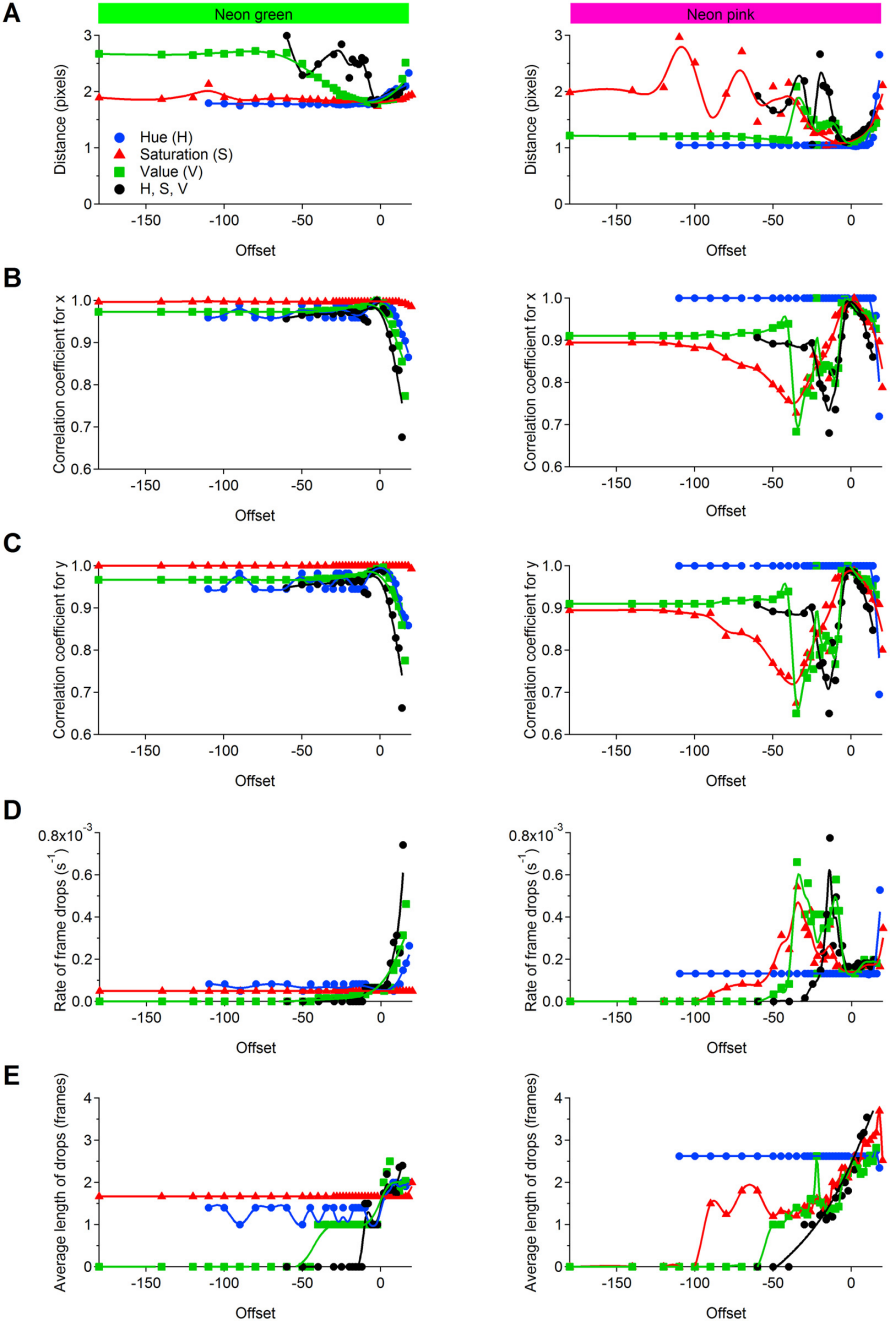
Accuracy of M-Track detection of forepaw position using different HSV settings. The data included in this figure were used to estimate how accurately M-Track detects the position of the forepaws labelled with different colors (Neon green and Neon magenta), using tight and looser limits for the H, S, or V parameters. The data shows the effect that loosening the HSV settings has on the detected position of the forepaws **(A)**, the correlation coefficient of the forepaw trajectories detected with optimal and offset HSV settings along the x **(B)** and y dimensions **(C)**, the number of frames dropped **(D)** and the length of time for which the frames are dropped **(E)**. Pixel size: 0.21 mm.

Loosely constraining the forepaws color masks reduces the accuracy with which M-Track detect the location of the forepaws. For example, M-Track can mistakenly track the forepaws when these go out of the field of view (e.g. behind the mouse head), ultimately giving inaccurate coordinates. It can also cause the algorithm to track other pixels that are not filtered by the color mask and that form clusters that are bigger than the selected “Min Box Size”. On the other hand, if the color mask is too tightly constrained, then any potential change in the background light of the behavioral arena or any other source of noise occurring during video acquisition may cause M-Track to incorrectly track or even lose the position of the forepaws in some frames (due to the fact that the color of the forepaw falls out of the set limits of the color mask). We established how loosely or tightly constraining the color masks affects the accurate detection of the position of the forepaws labeled with Neon green and Neon magenta (Fig 3). In Fig 3, negative offset values (*x-axis*) correspond to broader limits, positive offset values correspond to narrower limits. In general, when the HSV limits chosen to set the forepaws color masks are loosely constrained, M-Track provides a more accurate reading of the forepaw labelled with Neon green than the one labelled with Neon magenta. This causes M-Track to report a marginally different forepaws position. However, the error introduced in the position of the forepaws is negligible (i.e. <3 pixels, which corresponds to <0.63 mm in our experimental conditions) and unlikely to make a substantial contribution to estimates of total grooming distance. Consistent with these findings, the correlation coefficient between the position of the Neon green forepaw along the *x-* (Fig 3B, left) and *y-*axis (Fig 3C, left) using optimal and offset HSV settings remains high. The position of the forepaw labelled with Neon magenta is more prone to slight inaccuracies when offsetting the SV, not the H, limits (Fig 3B and 3C, right). Under these conditions, the inaccuracy arises from the fact that M-Track compares the frequency-count histogram in the reference ROI with the histogram of hue distribution in the visible pixels of a poorly constrained forepaw mask. The hue distribution in the non-masked pixels is unlikely to match with the one of the reference ROI, causing tracking errors. Despite these effects, the detected trajectories remain highly correlated with the ones detected with optimal HSV settings, due to the fact that the forepaw ROIs are small and the mean shift algorithm can still accurately determine the position where the forepaws are most likely to be in each video frame. Another effect of loosely constraining the HSV settings is that M-Track can eventually start dropping frames (i.e. it becomes unable to locate the forepaw position). The rate of frame drops is very small for the forepaw labelled with Neon green (Fig 3D, left) and slightly higher for the forepaw labeled with Neon magenta (Fig 3D, right). The length of time over which M-track loses the position of the forepaws is also shorter for the Neon green than the Neon magenta labelled (Fig 3E). As expected, broadening the detection limits for either the H, S or V parameters has different effects than doing so for all parameters at the same time (Fig 3). This is because changing the detection accuracy of parameters with a wide distribution of values has a more profound effect on the color mask than changing the detection accuracy of settings with very narrow distribution (which affect the color mask only within a narrow range). Detection settings that are too tightly constrained cause M-Track to track pixels that do not correspond to the forepaws, leading to errors in the detected trajectories and to more frequent and longer frame drops (Fig 3). It is important to note that despite the different susceptibility to errors for tracking the Neon green and Neon magenta forepaws, when the HSV parameters are well-constrained, M-Track detects both colors with similar accuracy. Therefore, the specific color used to label individual forepaws does not introduce any significant bias in the detection analysis as long as the user accurately constrains the corresponding HSV upper and lower limits in the M-Track GUI.

### Forepaw movement detection during grooming and walking, in black and white mice

**Fig 4:**
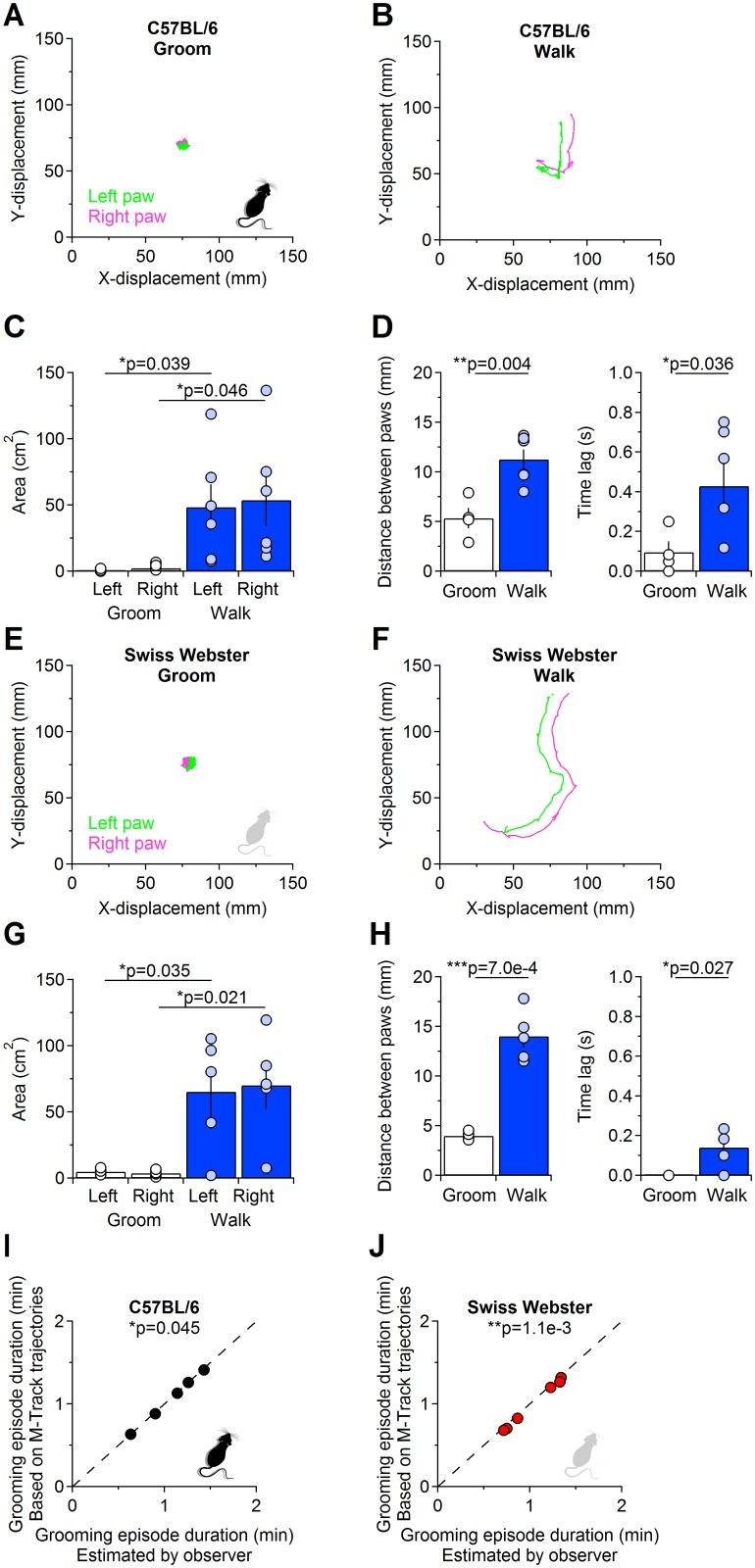
Example analysis of forepaw trajectories during grooming and walking. **(A)** Example of left (Neon green) and right (Neon magenta) forepaw trajectories detected using M-Track during a grooming episode in a C57BL/6 mouse (black fur). **(B)** As in A, detected during a walking episode. **(C)** Estimated surface area covered by the forepaws during grooming (left: 0.74±0.47 cm^2^, right: 2.20±0.83 cm^2^ (n = 4)) and walking (left: 48±17 cm^2^, right: 54±20 cm^2^ (n = 5), *p = 0.039 and *p = 0.046, respectively). **(D)** Left: the distance between the left and right forepaws is significantly longer during walking than during grooming episodes (grooming: 5.3±1.0 mm (n = 4), walking: 11.3±1.0 mm (n = 5), **p = 0.004). Right: the time lag in the movement of the left and right forepaws during grooming (white bars and hollow dots) is significantly shorter than that measured during walking episodes (blue bars and light blue dots; grooming: 0.10±0.05 s (n = 4), walking: 0.43±0.11 s (n = 5), *p = 0.036). **(E-H)** As in A-D, for a Swiss Webster mouse (white fur). **(I-J)** Relationship between the duration of grooming episodes calculated based on M-Track trajectories and based on the measures obtained by visual inspection of videos by an observer. The measures of grooming episode duration obtained using M-track are slightly but consistently shorter than the ones obtained based on visual inspection of the videos, in C57BL/6 (I; (n = 5) *p = 0.045) and Swiss Webster mice (J; (n = 6) **p = 1.1e-3).

**Fig 5:**
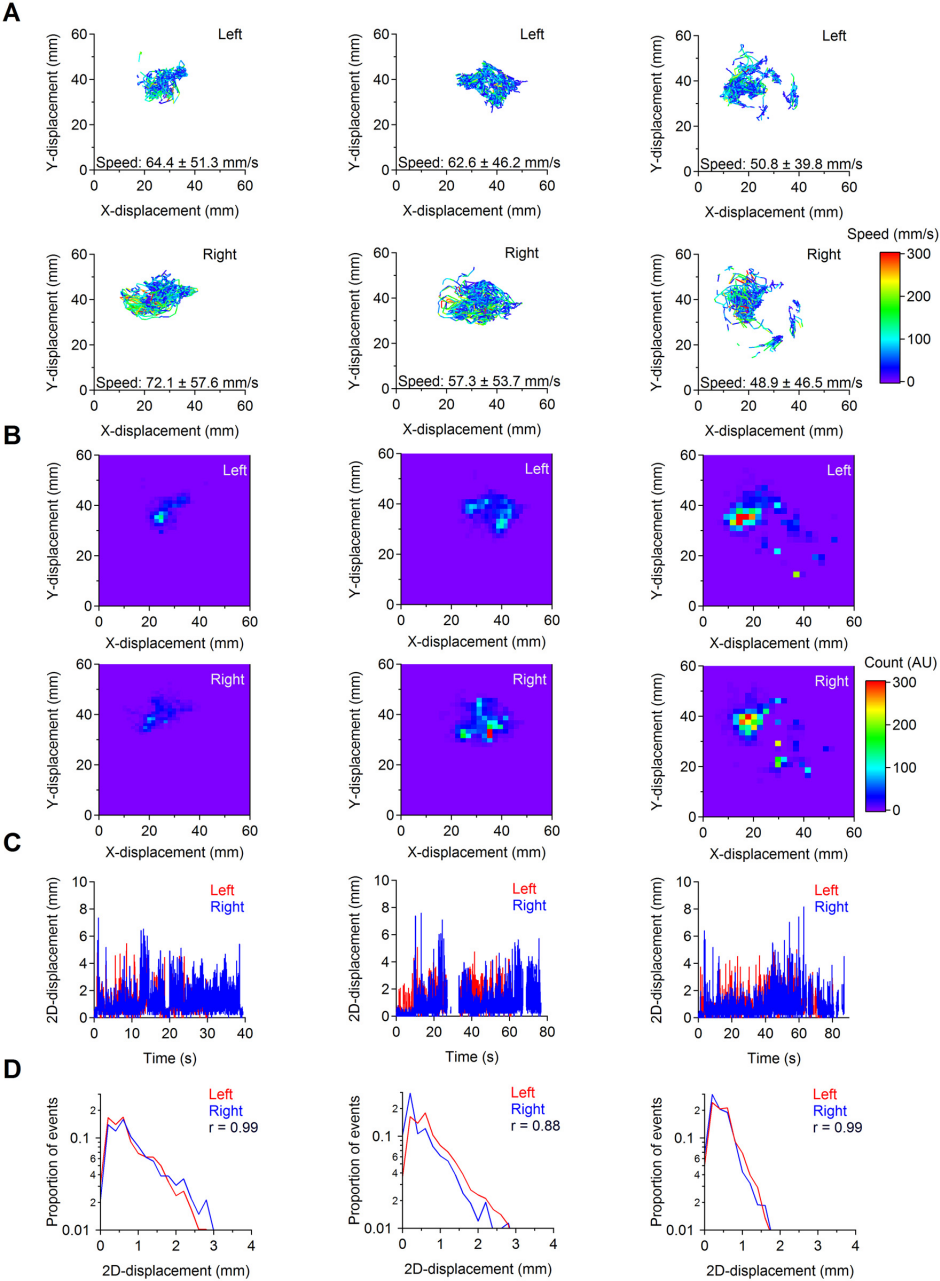
Example of forepaw trajectories during three consecutive grooming episodes. **(A)** Example of forepaw trajectories detected for the left (top) and right forepaw (bottom) during individual grooming episodes, using M-Track. The color coding of each trajectory represents the speed with which the forepaw is moved. Although the area of the body groomed varies across episodes, the average grooming speed remains similar for the left and right forepaws across grooming episodes. The speed values represent mean ± S.D. **(B)** Image plots obtained from grooming trajectories for the left (top) and right (bottom) forepaws. The color of the image represents the number of times that the trajectory of the forepaw has moved over a given pixel. **(C)** x,y-displacements for each forepaw during individual grooming episodes. **(D)** Frequency count histogram of the frame-to-frame displacements for the left and right forepaws. We observed a high level of correlation for each one of the recorded grooming episodes (Pearson’s correlation coefficient: r = 0.95±0.03 (n = 3)).

M-Track is specifically tailored to track the movement of forepaws during grooming episodes, but its robust detection algorithm makes it a valuable tool to also detect other types of movements (e.g. walking), in different strains of mice with different fur colors (e.g. C57BL/6, Swiss Webster). The accuracy of M-Track detection is similar in black and white mice, due to the fact that in the HSV color space black and white colors have the same hue (H = 0). In Fig 4, we show examples of grooming and walking trajectories detected by M-Track in C57BL6 (black fur; Fig 4A–4D and 4I) and Swiss Webster mice (white fur; Fig 4E–4H and 4J). Grooming and walking episodes can be distinguished because during grooming the forepaws move over a very confined area, which can be measured as the area of the grooming trajectory projected on the 2D field of view of the camera in C57BL/6 (left: 0.74±0.47 cm^2^, right: 2.20±0.83 cm^2^ (n = 4)) and Swiss Webster mice (left: 4.96±1.11 cm^2^, right: 3.70±1.21 cm^2^ (n = 4)). In contrast, during walking, the forepaws move over broader areas within the behavioral enclosure in C57BL/6 (left: 48±17 cm^2^, right: 54±20 cm^2^ (n = 5), *p = 0.039 and *p = 0.046, respectively; Fig 4A–4C) and Swiss Webster mice (left: 65±19 cm^2^, right: 70±18 cm^2^ (n = 6), *p = 0.035 and *p = 0.021, respectively; Fig 4E–4G). During grooming, the distance between the forepaws is small compared to the one measured during walking (C57BL/6 –grooming: 5.3±1.0 mm, walking: 11.3±1.0 mm, **p = 0.004; Swiss Webster—grooming: 4.0±0.2 mm, walking: 14.0±1.1 mm, **p = 7.0e-4; Fig 4D and 4H left). The movement of the two forepaws is correlated during both grooming and walking (see Fig 5) but the left-right alternation of the forfepaws during walking introduces a noticeable time lag between the movement of the left and right forepaws, which is not detected during grooming (C57BL/6 –time lag during grooming: 0.10±0.05 s, time lag during walking: 0.43±0.11 s, *p = 0.036; Swiss Webster—time lag during grooming: 0.00±0.00 s, time lag during walking: 0.14±0.04 s, *p = 0.027; Fig 4D and 4H right). Therefore, any of these criteria can be used to discriminate between grooming and walking based on the output data generated by M-Track. The grooming trajectories generated by M-Track also make it possible to obtain estimates of the duration of grooming episodes (Fig 4I and 4J). The duration of grooming episodes estimated based on M-Track trajectories is marginally, but consistently shorter than the one based on simple visual inspection of the videos (C57BL/6: M-Track 1.27±0.24 min, observer 1.29±0.24 min (n = 6), *p = 0.045; Swiss Webster: M-Track 1.00±0.12 min, observer 1.04±0.12 min (n = 5), **p = 1.1e-3). Based on these findings, we suggest that M-Track can be used as a versatile tool for a number of applications that require to accurately detect not only grooming but also and other types of movements, including walking, in mice with black or white fur.

### Forepaw movement analysis during grooming

The text output file generated by M-Track can be analyzed using a number of analysis software. We imported the M-Track text files in IgorPro 6.3.6.4 (Wavemetrics) and used custom-made routines to display and analyze the trajectories of right and left forepaws and built-in functions to generate color maps for the time spent by each forepaw in different regions of the behavioral enclosure. An example of left and right forepaws trajectories is shown in Fig 5, for three consecutive grooming episodes performed by the same C57BL/6 mouse. The trajectories shown in each panel correspond to the *x*,*y* position of the forepaws while the mouse grooms the mouth, nose and face (Fig 5A, left), head (Fig 5A, middle) and the rest of the body (Fig 5A, right). In this figure, the color coding represents the speed with which the mouse executes each movement, which is inversely proportional to the frame-by-frame *x*,*y* displacement of each forepaw. Notably, the speed with which each forepaw is moved does not change significantly as the mouse grooms different and broader parts of its body. The output file generated by M-Track also allows to visualize how the forepaws are moved during grooming and how much time is spent by each forepaw to groom a specific part of the mouse body (Fig 5B). By performing a frame-by-frame displacement analysis (Fig 5C), we estimated the correlation coefficient for the left and right paws during distinct grooming episodes. In the example shown in Fig 5D, the frame-by-frame displacement of the left and right forepaws remain similar across distinct grooming episodes.

**Fig 6:**
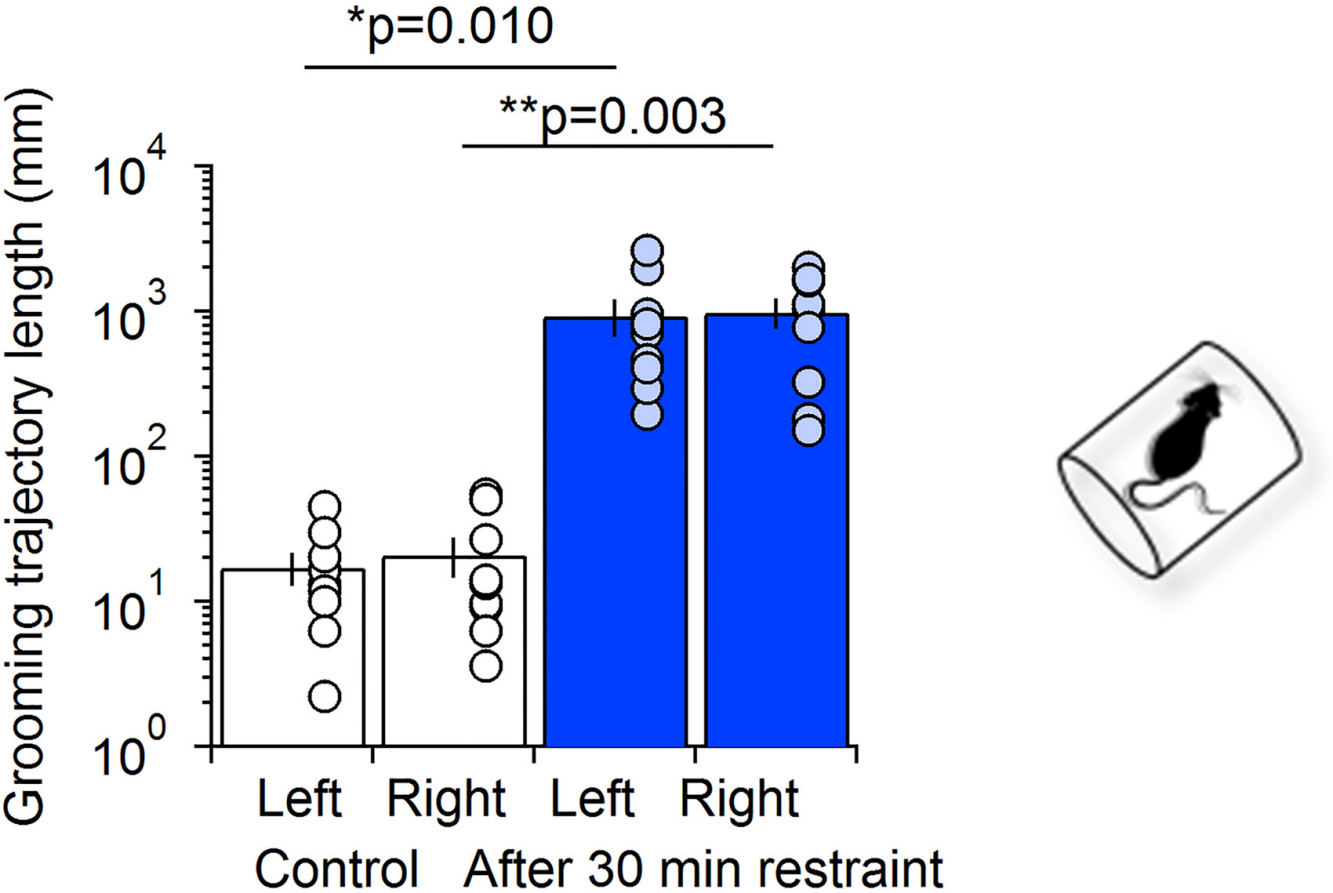
M-Track analysis of grooming trajectories in mice exposed to 30 min restraint stress. The M-Track analysis reveals that the grooming trajectory length of the left and right forepaws is significantly increased following exposure to restraint stress (Control: left forepaw 17.2 ± 4.4 mm, right forepaw 20.9 ± 6.4 mm (n = 9); Restraint: left forepaw 935.3 ± 273.3 mm, right forepaw 990.6 ± 229.6 mm (n = 9); left *p = 0.010, right **p = 0.003).

As a final test to validate the usefulness of the grooming trajectory analysis made possible by M-Track, we measured the total length of the grooming trajectory of the left and right forepaws in mice exposed to restraint stress (Fig 6). The data show that a 30 min exposure to restraint stress induces a profound increase in the total length of the grooming trajectory for the left and right forepaws (Control: left forepaw 17.2 ± 4.4 mm, right forepaw 20.9 ± 6.4 mm (n = 9); Restraint: left forepaw 935.3 ± 273.3 mm, right forepaw 990.6 ± 229.6 mm (n = 9); left *p = 0.010, right **p = 0.003). This finding is consistent with the data shown in Fig 1J–1L showing that restraint stress prolongs the duration of grooming episodes in mice.

## Availability and Future Directions

In this work we described a simple experimental set-up and a new open source software, M-Track, which can be used and implemented to analyze spontaneous and evoked grooming (and walking) in freely-moving mice. The software and documentation for M-Track is freely available for download from the Scimemi Lab website (https://sites.google.com/site/scimemilab2013/software) or from the GitHub repository, which also includes detailed instructions on software installation and video analysis, which can be tested in videos of black and white mice (https://github.com/scimemia/M-Track). The development of this software fills important gaps in the analysis of mouse behavior. Grooming is a complex and common innate behavior performed by mice. The time spent grooming and the stereotypy with which patterned movements are executed during grooming episodes carry invaluable information about proper function of neural circuits implicated with the execution of stereotyped activities and are altered in response to stress exposure, during pharmacological manipulation of dopaminergic circuits and in animal models of autism spectrum disorders [10, 28]. Despite its known relevance for behavioral neuroscience studies, the analysis of grooming for numerous investigators still relies on visual monitoring and manual scoring of individual grooming episodes. Automated tools to analyze animal behavior have become increasingly popular, but none of them has been specifically tailored to analyze grooming in freely-behaving mice not exposed to stress [6, 8, 21, 23]. M-Track provides a user-friendly GUI and robust algorithm to accurately track the movement of individual forepaws during spontaneous grooming in freely-behaving mice. The forepaw detection algorithm used by M-track is ideally tailored to unambiguously track the forepaws movement even in video files where the forepaws transiently disappear from the field of view during head and body grooming. There are many exciting applications for analyzing the output data generated by M-Track. For example, they can provide information on the speed with which grooming episodes are performed, the area of the body that each mouse covers during distinct grooming episodes, the level of bilateral coordination of the two forepaws during grooming and the total grooming trajectory. This new information can help extend the grooming analysis in a number of laboratories beyond simple inspection of grooming frequency and total grooming time. Although M-Track was originally design to detect grooming, it can also be used to track other types of movements, including walking. We believe M-Track will be particularly useful to the behavioral neuroscience community for automated behavior classification of complex grooming behaviors in animal models of neuropsychiatric disorders like obsessive compulsive disorder and Tourette’s syndrome. We plan to further implement the M-Track software with new detection algorithms and analysis routines, to broaden the accessibility of grooming analysis to the behavioral neuroscience community. Users are also encouraged to generate their own bundled algorithms and make them publicly available through the M-Track webpage, to help transparency and encourage cross-disciplinary collaborations.

## Materials and Methods

### Ethics statement

The video-tracking experiments were performed on C57BL/6 and Swiss Webster mice of either sex (1–4 months old). All experiment protocols were performed in accordance with the guidelines and were approved by the Institutional Animal Care and Use Committee at SUNY Albany. The restraint stress was performed by keeping the mice in a ventilated restraint tube for 30 min. Neon green and Neon magenta permanent markers (Sharpie) were used to label the right and left forepaws of each mouse. We applied 2–4 layers of color every 2–3 min. All videos used for trajectory analysis were acquired 20 min after applying the last layer of color, unless otherwise stated. The Sharpie markers typically start fading ~40 min after being applied to the mouse forepaws and are largely gone after two or more hours after being applied. Unless otherwise stated, the beginning and end point of a grooming episode was determine by the experimenter during video analysis. We used a white neon light as a mild stressor for mice (14,000 lux; Fig 1J–1L). The light was positioned 10” above the behavioral arena and was kept on for 5 min. No change in the temperature of the behavioral arena (20°C) was detected over the course of the light stress exposure, confirming that the detected effects could not be attributed to heat stress. All data are presented as mean ± S.E.M, unless otherwise stated.

### Assembly equipment for the behavioral arena

The behavioral arena used to perform the video tracking analysis consisted of four grooming chambers with clear bottom and white side walls. All materials required to build the grooming chamber are reported in Table 1.

**Table 1:** 

*Item Name*	*Quantity*	*Manufacturer*	*Model*
Light bulbs	2	Feit Electric	60W Equivalent black spiral CFL light bulb
Light bulbs	2	EcoSmart	60W Equivalent daylight spiral CFL light bulb
Markers	2	Sharpie	Neon green, Neon magenta
White Plexiglas (1/8” thick)	1	Amazon	12”x20”
	2	Amazon	12”x6”
	2	Amazon	20”x6”
Clear acrylic sheet	1	EPlastics	12”x20”x1/32”
Extruded Acrylic Tube	4	EPlastics	1”x1”x 4”

### Video acquisition

A digital SLR camera was positioned below the grooming chamber to monitor the grooming behavior of each mouse. The camera and memory card specifications are reported in Table 2.

**Table 2:** 

*Item Name*	*Quantity*	*Manufacturer*	*Model*
Camera	1	Canon	EOS Rebel T3i Digital SLR Camera with EF-S 18–55 mm f/3.5–5.6 IS Lens
Memory Card	1	ScanDisk	16 GB HD SC Memory Card

### Coding

M-Track was written using Python 2.7, OpenCV 3.0 and Qt4.8, which can be downloaded free of charge at the URLs reported in Table 3.

**Table 3:** 

*Item Name*	*URL Link*
Python 2.7	https://www.python.org/download/releases/2.7/
OpenCV 3.0	http://opencv.org/downloads.html
Qt4.8	https://download.qt.io/archive/qt/4.8/4.8.5/

### Software

The software and documentation for M-Track are freely available for download from the Scimemi lab website (https://sites.google.com/site/scimemilab2013/software) or from the GitHub repository (https://github.com/scimemia/M-Track). M-Track is available as Microsoft Windows, Linux and Mac OS X source code written using Python 2.7, OpenCV 3.0 and Qt4.8. We provide two standalone versions of M-Track compatible with Microsoft Windows and Linux operating systems, with and without visualization of mouse body orientation. M-Track analyzes 8-bit grayscale StreamPix (Norpix Sequence Format: seq), .TIFF, .MPEG, .MOV and .AVI formatted video files. The output text file generated by M-Track was analyzed in IgorPro 6.3.6.4 (Wavemetrics) using custom-made routines (A.S.).
